# Significance of training, monitoring and assessment of malaria workers in achieving malaria elimination goal of Malaria Elimination Demonstration Project

**DOI:** 10.1186/s12936-020-03534-9

**Published:** 2021-01-07

**Authors:** Harsh Rajvanshi, Sekh Nisar, Praveen K. Bharti, Himanshu Jayswar, Ashok K. Mishra, Ravendra K. Sharma, Kalyan B. Saha, Man Mohan Shukla, Aparup Das, Harpreet Kaur, Suman L. Wattal, Altaf A. Lal

**Affiliations:** 1 Malaria Elimination Demonstration Project, Mandla, Madhya Pradesh India; 2 Indian Council of Medical Research-National Institute of Research in Tribal Health (ICMR-NIRTH), Jabalpur, Madhya Pradesh India; 3 Directorate of Health Services, Government of Madhya Pradesh, Bhopal, India; 4 Indian Council of Medical Research, Department of Health Research, Ministry of Health and Family Welfare, New Delhi, India; 5 Ministry of Health and Family Welfare, National Vector Borne Disease Control Programme, New Delhi, India; 6 Foundation for Disease Elimination and Control of India, Mumbai, Maharashtra India

## Abstract

**Background:**

The Malaria Elimination Demonstration Project (MEDP) maintained a workforce of 235 Village Malaria Workers (VMWs) and 25 Malaria Field Coordinators (MFCs) to conduct disease surveillance, case management, IEC/BCC activities, capacity building, and monitoring of vector control activities in 1233 villages of Mandla, a high malaria endemic district of Madhya Pradesh in central India.

**Methods:**

The induction training was conducted for 3 days on malaria diagnosis, treatment, prevention, and ethics. All trainings were assessed using a pre and post-training assessment questionnaire, with 70% marks as qualifying threshold. The questionnaire was divided into three thematic areas viz*.* general knowledge related to malaria (KAP), diagnosis and treatment (DXRX), and vector control (PVC).

**Results:**

In 2017, the project trained 330 candidates, followed by 243 and 247 candidates in 2018 and 2019, respectively. 94.3% candidates passed after a single training session. Almost all (95%) candidates showed improvement in knowledge after the training with 4% showing no effect and 1% showing deterioration. Progressive improvement in scores of 2017 cohort was seen along with significant improvement in performance of candidates in 2019 after the introduction of systematic monitoring and ‘shadowing’ training exercises.

**Conclusion:**

The project has successfully demonstrated the value of recruitment of workers from the study area, outcome of training, and performance evaluation of field staff in malaria elimination programme. This careful strategy of recruitment and training resulted in a work-force that was capable of independently conducting surveillance, case management, vector control, and Information Education Communication/Behaviour Change Communication (IEC/BCC). The learnings of this study, including the training modules and monitoring processes, can be used to train the health delivery staff for achieving national goal for malaria elimination by 2030. Similar training and monitoring programmes could also be used for other public health delivery programmes.

## Background

In the year 2019, the WHO South-East Asia region witnessed a reduction in malaria case incidence by 78%. India contributed to largest absolute reduction in cases from 20 million cases in 2000 to about 5.6 million cases in 2019. The global incidence rate has dropped down from 80 cases in 2000 to 56 cases per 1000 population at risk in the year 2019 [1]. There are several factors responsible for the gains in malaria control and elimination throughout the world, one of them is well-trained human resource at the field level that conduct surveillance, provide vector control tools, and perform rapid diagnosis for prompt treatment of malaria. The prevailing COVID-19 pandemic has posed additional challenges, because of the risk of moving funding and human resources away from the malaria elimination programme for pandemic preparedness and response work [2–4].

Irrespective of the challenges imposed on the health delivery systems, it has been observed that the trained human resource component is a key challenge in malaria elimination [5–7]. In India, the field staff is primarily responsible for fever surveillance and case management along with added responsibilities of spreading awareness about disease, referring serious cases to facilities, and monitoring vector control activities. Ground implementation involves physically covering the entire target geographical area on a regular basis for active surveillance-based treatment of malaria. This is a resource intensive exercise, which is the reason for the human resources to be a major cost of the public health programmes. Planning, recruitment, training, assessments, execution, monitoring, feedback, course-correction plays a crucial role [8, 9]. Assessment of these workers is critical to achieve malaria elimination goals throughout the world [10–13].

Malaria Elimination Demonstration Project is operational in the tribal district of Mandla in the state of Madhya Pradesh sprawling over 8770 sq. Kms with 1.16 million population since 2017. The project uses active case detection as part of its surveillance and case management strategy. This involves door-to-door visits to track fever, followed by testing, treatment, and tracking of treated patients. For better community acceptance, preference is always given for recruitment of field workers from the local areas. However, it has been difficult to find candidates with appropriate educational qualifications, training, or knowledge of malaria. In order to overcome this problem, a training programme was instituted as part of on-boarding of field staff. The recruited Village Malaria Workers (VMWs) and Malaria Field Coordinators (MFCs) were trained in fever surveillance and case management, vector control, IEC/BCC, and capacity building [14, 15]. To ensure that the recruited staff retained information about malaria throughout the conduct of the project, refresher training programmes comparable to the Continuing Medical Education (CMEs) were instituted for the field staff. These CMEs were designed so that the field workers become trained for recruitment by other public health programmes after they finish their assignment with MEDP.

For the recruitment of 422 field staff during 2017–2019, over 2200 candidates have been taken through selection process using written test (general knowledge and aptitude test), and two levels of interviews. The objective of the present study was to assess the effectiveness of recruitment and induction trainings by comparing knowledge of malaria through pre- and post-training performance evaluation of the recruited field staff.

## Methods

The recruitment of the field staff for the Malaria Elimination Demonstration Project (MEDP) was done from within the district. Applications were invited using newspaper advertisements, word-of-mouth, and social media platforms. Minimum age and educational criteria were 18 years and intermediate (class 12th), respectively. The recruitment was completed using a three-tier system. First tier involved screening using a general knowledge assessment of the candidates. Candidates who cleared the screening (50% qualifying threshold) moved to the second tier, which involved personal interview by the District Officer. After clearing this interview, candidates appeared for the third tier, which was the final personal interview by the Project Director or the Programme Officer [15]. Following the screenings, candidates were provided a choice of posting for a place near their home village. Successful candidates were provisionally recruited into the project, subject to successful completion of the induction trainings. The refresher trainings were conducted in the middle of the calendar year (June–July) each year, whereas, the induction training was conducted only once. Each VMW had the responsibility of door-to-door active surveillance in 6–8 villages. Each household was visited within an interval of 7–14 days depending upon the size and population of the working area. These VMWs were supervised by 25 MFCs, who were posted at the cluster-level.

The candidates appearing for the trainings were administered a 20-questions pre- and post-training assessment examination (Additional file 1: Annexure S1). The examination was divided into three thematic areas with the 20 questions divided amongst them viz*.* general knowledge related to malaria (KAP—8 questions), diagnosis and treatment (DXRX—8 questions), and vector control (PVC—4 questions) (Table 1). The maximum marks for three areas were same as the number of questions. These three thematic areas were chosen because of their relevance to malaria elimination programme.

**Table 1:** General socio-demographic information of the candidates trained under MEDP study in Mandla district, Madhya Pradesh

	2017 candidate	2018 candidates	2019 candidates
	% (n)	% (n)	% (n)
Age distribution ( years)			
≤ 25	33.92 (112)	29.63 (72)	37.77 (156)
25–35	54.24 (179)	56.79 (138)	48.18 (199)
36+	11.82 (39)	13.58 (33)	14.04 (58)
Total	100 (330)	100 (243)	100 (413)
Gender			
Male	83.03 (274)	83.13 (202)	83.05 (343)
Female	16.97 (56)	16.87 (41)	16.95 (70)
Total	100 (330)	100 (243)	100 (413)
Education			
Class 12	46.06 (152)	37.45 (91)	27.36 (113)
Graduate and above	53.94 (178)	62.55 (152)	72.64 (300)
Total	100 (330)	100 (243)	100 (413)
Caste			
General category	9.7 (32)	8.64 (21)	6.54 (27)
Other Backward Castes (OBC)	50 (165)	53.91 (131)	56.42 (233)
Scheduled Castes (SC)	14.24 (47)	14.81 (36)	15.01 (62)
Scheduled Tribes (ST)	26.06 (86)	22.63 (55)	22.03 (91)
Total	100 (330)	100 (243)	100 (413)

### Data analysis

The pre-training scores of the year 2017 were considered as the baseline. To assess the impact of the training, post-training scores the candidates from 2017 cohort were used. This cohort had 330 candidates in 2017, 212 candidates in 2018, and 166 candidates in 2019. The quantitative data was entered in CS PRO. The qualitative data was examined and converted to quantitative data using thematic analysis. For instance, in Q18 (Annexure 1), if the candidate answered full names of the malaria parasites (*Plasmodium falciparum*,* Plasmodium vivax*), she/he was awarded full marks, however, for incomplete answers like *falciparum*,* vivax*, only 50% marks were awarded. Following which, the data was analysed using IBM-SPSS version 23.0 for performing descriptive, bivariate, paired student t-test, and ANOVA statistical tests.

## Results

**Fig. 1:**
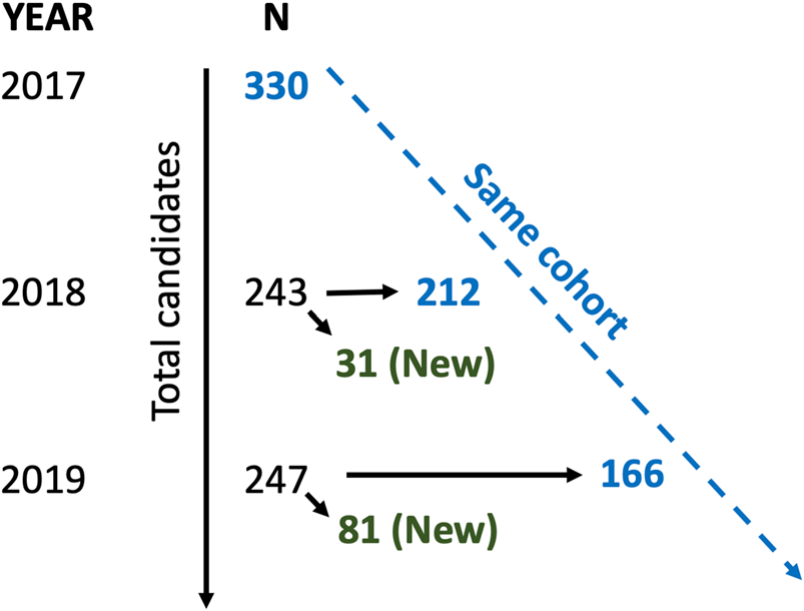
Number of candidates in each year of trainings. The total candidates in each year consisted of fresh candidates appearing for the first time in the assessment (in green colour) and old candidates from the 2017 cohort (in blue colour)

Since 2017, a total of 14 training sessions were conducted. In the year 2017, 330 candidates received the induction (on-boarding) training. In the year 2018, 243 candidates received the trainings, out of which 212 were already working on the project and were brought in for refresher training. The remaining 31 candidates were the new hires. Similarly, in 2019, out of the 247 candidates, 166 belonged to the cohort of 2017, and 81 candidates appeared for the first time and participated in the induction trainings (Fig. 1).

In the year 2017, out of 330 candidates, 33.9% (112) were less than or equal to 25 years of age, 54.2% (179) were 26 to 35 years of age, and 11.8% (39) were more than 36 years of age. Male candidates constituted 83% (274) and females 17% (56) of the candidates. Educational status showed that 46.1% (152) candidates had cleared 12th grade and 53.9% (178) were college graduates (3–4 years of college after 12th grade). Caste distribution revealed 9.7% (32) candidates from general category at 9.7% (32), 50% (165) from Other Backward Castes (OBS), 14.2% (47) from Scheduled Castes (SC) 14.2%, and 26.1% (86) from Scheduled Tribes (ST).

Till 2019, a total of 442 candidates were trained with a yearly attrition rate of 35.7% in 2017–2018 and 21.6% in 2018–2019. For the candidates trained, a passing percentage of 94.3% was achieved after a single session of trainings. Improvement in scores was seen in 420 (95%) candidates post-training. Total of 18 candidates (4%) showed no improvement and four candidates (1%) showed deterioration in scores in the post-training examination.

**Fig. 2:**
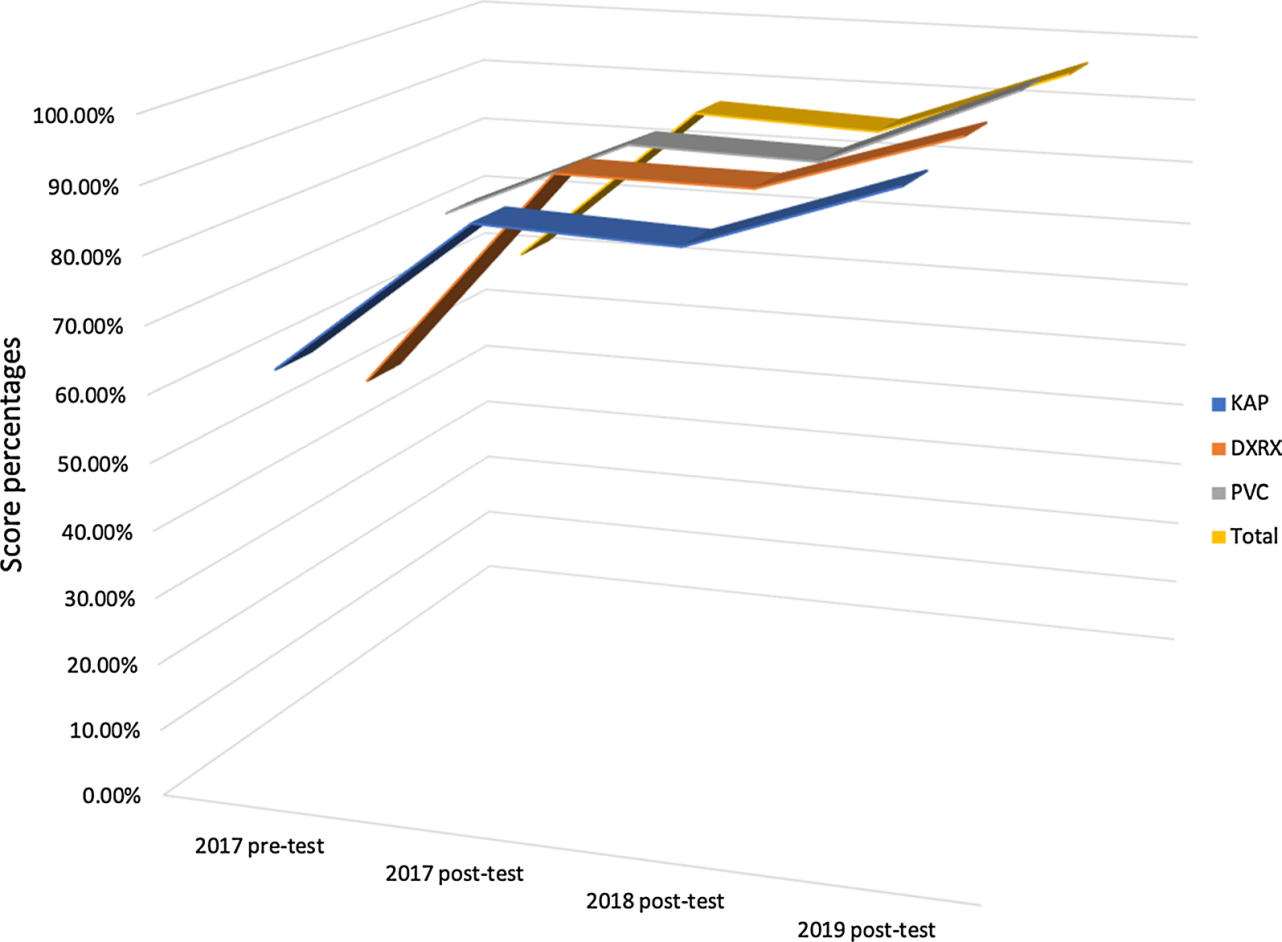
Comparison of scores from 2017 to 2019 in all three thematic areas along with total scores. All values are in percentages. The yellow line indicates total mean scores (addition of all thematic areas), orange line indicates the diagnosis and treatment scores (DXRX), blue line indicates the knowledge, attitude, and perception scores (KAP), and the grey line indicates the prevention and vector control scores (PVC)

The mean total score in pre-training examination was 12.29 against a maximum score of 20. The mean total score in post-training was 17.36. Upon further segregation of scores into the three thematic areas of general knowledge related to malaria (KAP), diagnosis and treatment (DXRX), and vector control (PVC) showed statistically significant improvement in scores as demonstrated in the post-training examinations, which reflects that continuous training programmes have a place in maintaining the skillsets of field staff (Fig. 2).

A bivariate analysis of the pre- and post-training scores in the three thematic areas was done using age, gender, educational status, and caste as variables. There was no statistical difference between the pre- and post-training scores of candidates when compared amongst different age groups (≤ 25 years, 26 to 35 years, 36+ years), between male and female candidates, between 12th pass and graduates, and amongst different caste groups. Males scored higher (12.38) than females (11.86) in pre-training but less in post-training (17.33) than females (17.52). Graduates scored higher in both pre- and post-training scores than 12th grade candidates, but the difference in both groups was not statistically significant. Tables 2, 3, 4, 5 shows scores from 2017 to 2019 for old cohort (2017) and new candidates (2018 and 2019) using bivariate analysis according to gender, age, caste, and educational qualification.

**Table 2:** Gender-wise comparison of mean scores of candidates trained under MEDP study across different years

Year	Female					Male					p-value
	% (n)	Mean score				% (n)	Mean score				
		KAP	DXRX	PVC	Total		KAP	DXRX	PVC	Total	
2017 (pre-training)											
Fresh candidates	16.97 (56)	61.38 (4.91)	51.38 (4.11)	71 (2.84)	59.30 (11.86)	83.03 (274)	63 (5.04)	54.50 (4.36)	74.75 (2.99)	61.90 (12.38)	0.17
2017 (post-training)											
Fresh candidates	16.97 (56)	86.63 (6.93)	90.63 (7.25)	83.50 (3.34)	87.60 (17.52)	83.03 (274)	86 (6.88)	87 (6.96)	87.25 (3.49)	86.65 (17.33)	0.5
2018 (post-training)											
Fresh candidates	1.23 (3)	79.17 (6.33)	80.83 (6.46)	70.83 (2.83)	78.17 (15.63)	11.52 (28)	83.04 (6.64)	85 (6.8)	85.72 (3.42)	84.36 (16.87)	0.5
2017 cohort	15.64 (38)	86.84 (6.94)	91.25 (7.3)	92.76 (3.71)	89.79 (17.95)	71.60 (174)	87.07 (6.96)	89.89 (7.19)	86.93 (3.47)	88.17 (17.63)	0.2
2019 (post-training)											
Fresh candidates	9.93 (41)	95.21 (7.61)	96.91 (7.75)	98.79 (3.95)	96.60 (19.32)	49.88 (206)	95.09 (7.60)	96.66 (7.73)	98.48 (3.93)	96.40 (19.27)	0.8
2017 cohort	7.02 (29)	95.53 (7.64)	97.35 (7.78)	98.72 (3.94)	96.90 (19.37)	33.17 (137)	95.25 (7.62)	96.88 (7.75)	98.25 (3.93)	96.50 (19.3)	0.8

**Table 3:** Age-wise comparison of mean scores of candidates trained under MEDP study across different years

Year	≤ 25 years					25–35 years					35+ years					p-value
	% (n)	Mean score				% (n)	mean score				% (n)	mean score				
		KAP	DXRX	PVC	Total		KAP	DXRX	PVC	Total		KAP	DXRX	PVC	Total	
2017 (pre-training)																
Fresh candidates	33.94 (112)	64.13 (5.13)	53.25 (4.26)	72.75 (2.91)	61.40 (12.28)	54.24 (179)	62.38 (4.99)	53.50 (4.28)	74.50 (2.98)	61.25 (12.25)	11.82 (39)	60.25 (4.82)	58 (4.64)	76.25 (3.05)	62.55 (12.51)	0.62
2017 (post-training)																
Fresh candidates	33.94 (112)	87 (6.96)	88.25 (7.06)	88.50 (3.54)	87.80 (17.56)	54.24 (179)	85.13 (6.81)	87.38 (6.99)	85 (3.4)	86.05 (17.21)	11.82 (39)	87.88 (7.03)	86.88 (6.95)	88.50 (3.54)	87.55 (17.51)	0.39
2018 (post-training)																
Fresh candidates	0.82 (2)	68.75 (5.5)	85 (6.8)	87.50 (3.5)	84 (16.8)	9.47 (23)	84.75 (6.78)	86.63 (6.93)	86.50 (3.46)	85.85 (17.17)	2.47 (6)	79.17 (6.33)	76.67 (6.13)	66.67 (2.66)	75.67 (15.13)	0.04
2017 cohort	28.81 (70)	90.71 (7.25)	92.68 (7.41)	93.57 (3.74)	92.07 (18.41)	47.33 (115)	84.67 (6.77)	89.35 (7.14)	85.54 (3.42)	86.72 (17.34)	11.11 (27)	87.50 (7)	86.85 (6.94)	83.80 (3.35)	86.50 (17.3)	0.04
2019 (post- training)																
Fresh candidates	23.73 (98)	95.50 (7.64)	96.88 (7.75)	99.25 (3.97)	96.85 (19.37)	28.33 (117)	93.75 (7.5)	97.66 (7.81)	97.66 (3.90)	96.09 (19.21)	7.75 (32)	95.13 (7.61)	96.63 (7.73)	98.50 (3.94)	96.40 (19.28)	0.6
2017 cohort	14.04 (58)	95.88 (7.67)	97.10 (7.76)	99.09 (3.96)	97 (19.40)	19.85 (82)	92.79 (7.42)	97.12 (7.76)	97.12 (3.88)	95.38 (19.07)	6.30 (26)	95.26 (7.62)	96.84 (7.74)	98.34 (3.93)	96.51 (19.30)	0.6

**Table 4:** Caste-wise comparison of mean scores of candidates trained under MEDP study across different years

Year	General category					Other Backward Castes					Scheduled Castes					Scheduled Tribes					p-value
	% (n)	Mean score				% (n)	Mean score				% (n)	Mean score				% (n)	Mean score				
		KAP	DXRX	PVC	Total		KAP	DXRX	PVC	Total		KAP	DXRX	PVC	Total		KAP	DXRX	PVC	Total	
2017 (pre- training)																					
Fresh candidates	9.7 (32)	66.38 (5.31)	51.13 (4.09)	72.75 (2.91)	61.55 (12.31)	50 (165)	64.25 (5.14)	54.13 (4.33)	72.5 (2.9)	61.75 (12.35)	14.24 (47)	55.88 (4.47)	59 (4.72)	73.5 (2.94)	60.65 (12.13)	26.06 (86)	62.38 (4.99)	51.88 (4.15)	78.25 (3.13)	61.35 (12.27)	0.95
2017 (post- training)																					
Fresh candidates	9.7 (32)	87.88 (7.03)	89.13 (7.13)	89.75 (3.59)	88.75 (17.75)	50 (165)	85.5 (6.84)	87.63 (7.01)	88 (3.52)	86.8 (17.36)	14.24 (47)	89.63 (7.17)	88 (7.04)	84.5 (3.38)	88 (17.6)	26.06 (86)	84.63 (6.77)	87.13 (6.97)	84 (3.36)	85.45 (17.09)	0.31
2018 (post-training)																					
Fresh candidates	0.06 (2)	81.25 (6.5)	97.50 (7.8)	87.50 (3.5)	89 (17.8)	6.17 (15)	87.5 (7)	88.33 (7.06)	87.5 (3.5)	87.83 (17.56)	2.06 (5)	82.5 (6.6)	85 (6.8)	92.5 (3.7)	85.5 (17.1)	3.7 (9)	75 (6)	75.28 (6.02)	73.61 (2.94)	74.83 (14.96)	0.08
2017 cohort	7.82 (19)	94.74 (7.57)	98.82 (7.90)	95 (3.8)	98.08 (19.61)	47.74 (116)	86.10 (6.88)	90.60 (7.24)	88.58 (3.54)	88.4 (17.67)	12.76 (31)	89.92 (7.19)	88.23 (7.05)	86.69 (3.46)	88.6 (17.71)	18.93 (46)	84.24 (6.73)	86.63 (6.93)	80.98 (3.23)	84.54 (16.90)	0.06
2019 (post-training)																					
Fresh candidates	3.87 (16)	94.89 (7.59)	96.53 (7.72)	98.54 (3.94)	96.28 (19.25)	33.17 (137)	97.25 (7.78)	97 (7.76)	99.25 (3.97)	97.55 (19.51)	8.96 (37)	93.64 (7.49)	96.27 (7.70)	97.37 (3.89)	95.44 (19.08)	12.8 (57)	95.09 (7.60)	96.66 (7.73)	98.48 (3.93)	96.4 (19.27)	0.57
2017 cohort	2.66 (11)	94.53 (7.56)	96.22 (7.69)	97.92 (3.91)	95.89 (19.17)	23.24 (96)	98 (7.84)	97 (7.76)	100 (4)	98 (19.6)	6.05 (25)	94.12 (7.52)	97.79 (7.82)	97.8 (3.91)	96.32 (19.26)	8.23 (34)	95.26 (7.62)	96.84 (7.74)	98.34 (3.93)	96.51 (19.30)	0.67

**Table 5:** Educational qualification-wise comparison of mean scores of candidates trained under MEDP study across different years

Year	12th pass					Graduate and above					p-value
	% (n)	Mean score				% (n)	Mean score				
		KAP	DXRX	PVC	Total		KAP	DXRX	PVC	Total	
2017 (pre-training)											
Fresh candidates	46.06 (152)	62.2 (4.98)	53 (4.24)	73.75 (2.95)	60.85 (12.17)	53.94 (178)	63.25 (5.06)	54.75 (4.38)	74.5 (2.98)	61.95 (12.39)	0.44
2017 (post-training)											
Fresh candidates	46.06 (152)	85.13 (6.81)	86.63 (6.93)	85.25 (3.41)	85.75 (17.15)	53.94 (178)	86.88 (6.95)	88.5 (7.08)	87.75 (3.51)	87.7 (17.54)	0.07
2018 (post-training)											
Fresh candidates	3.7 (9)	77.78 (6.22)	68.33 (5.46)	76.39 (3.05)	73.72 (14.74)	9.05 (22)	84.63 (6.77)	91.25 (7.3)	87.5 (3.5)	87.85 (17.57)	0.05
2017 cohort	33.74 (82)	84.91 (6.79)	88.66 (7.09)	86.59 (3.46)	86.74 (17.34)	53.5 (130)	88.37 (7.06)	91.06 (7.28)	88.85 (3.55)	89.54 (17.90)	0.24
2019 (post-training)											
Fresh candidates	15.98 (66)	95.51 (7.64)	96.75 (7.74)	99.17 (3.96)	96.74 (19.34)	43.83 (181)	95.09 (7.60)	96.66 (7.73)	98.48 (3.93)	96.4 (19.27)	0.7
2017 cohort	11.38 (47)	92.55 (7.40)	95.75 (7.65)	95.75 (3.82)	94.47 (18.89)	28.81 (119)	96.32 (7.70)	97.27 (7.78)	99.37 (3.97)	97.31 (19.46)	0.06

In the year 2018, a second round of training was conducted for the field staff. A total of 243 candidates were trained, out of which 212 candidates belonged to the 2017 cohort. First time trainings in 2018 were given to the remaining 31 candidates. Slight improvement in the mean scores of the 2017 cohort was observed. Upon comparison of the post-training scores of 31 candidates of 2018 against the scores of 2017 cohort who had received two trainings, the older cohort scored higher in all three thematic areas with no statistically significant difference between both the groups.

The bivariate analysis of the fresh candidates of 2018 revealed similar findings to post-training scores of 2017 with college graduates scoring higher than 12th pass, and general category scoring highest followed by OBC, SC, and ST. The only difference was that the age group of 25 to 35 years scored more this year as compared to the less than 35 years. age group scoring higher in 2017. But in older cohort of 2017 displayed similar patterns in age-group-specific performance in 2018 as well. Another notable finding was the females amongst the fresh candidates of 2018 scored lower than the male counterparts, but higher amongst the old 2017 cohort, which was same pattern as seen during 2017 trainings.

The difference between the mean scores of 12th grade and graduates in fresh candidates of 2018 was 2.83 marks. However, this difference was only 0.56 marks in the older 2017 cohort. Upon further investigation, the difference was of only one mark amongst the fresh candidates of 2018 in the 2019 post-training scores. Similar patterns were noticed in subsequent trainings.

Third round of trainings was conducted in 2019. A total of 247 candidates participated in the training, out of which 166 candidates belonged to the 2017 cohort and were appearing in the training for the third time. First time trainings were given to 81 candidates. This year, the mean total score of both first-time trainees and 2017 cohort was 19.3 out of a maximum score of 20, with statistically significant difference (p < 0.05) against the scores of preceding years (Fig. 2). Similar improvement was seen in all three individual thematic areas. Bivariate analysis against age group, gender, educational status, and caste also revealed significant improvement in scores as compared to the 2017 post-training and 2018 post-training scores of the candidates. It was noted that not only 2017 cohort candidates who have received three trainings scored better, but all the fresh candidates appearing for the first time in 2019 also scored better than the fresh candidates of preceding years, but slightly less than 2017 cohort.

In 2019 trainings, the female field staff of both 2017 cohort and the fresh batch of 2019 scored higher than males in the post-training. It was noted note that 12th grade candidates scored slightly higher than the graduates in the new 2019 cohort. It was also noted that the candidates of 2018 cohort scored higher than graduates in the 2019 post-training assessment. Change in order of scores amongst different castes was seen in fresh candidates of 2019 and 2017 cohort, this time all of them scored more than 19 out of 20.

The qualifying marks for Village Malaria Workers as part of MEDP were minimum 70%. In the year 2017, most of the candidates qualified (88%) in a single attempt. In the year 2018, this number rose to 95%, and after effective implementation of monitoring checklists (Additional file 2: Annexure S2) and shadowing, this number rose to 100% in the 2019 batch of trainees.

## Discussion

This study presents findings of training on knowledge of Village Malaria Workers recruited to conduct work on the Malaria Elimination Demonstration Project. Globally, the importance of training of malaria health workers has been considered a high-priority component to achieve malaria elimination [16–19]. A qualitative analysis of various training studies done by Atkinson et al*.* has highlighted the need for adequate training of locally recruited volunteers [16]. The experience from China’s malaria elimination has also highlighted the need for more on-hands training for peripheral malaria workers [17]. The current study compliments the global advisory of the need for high-quality training for the field workers for malaria elimination [18, 19]. Since the field workers would be most likely recruited on a temporary basis, these training especially important to groom them for other public health programmes of national importance.

The refresher trainings were conducted for the entire field staff with a goal to continuously inform them on surveillance, case management, and vector control components of malaria elimination, as well as to introduce any new diagnostic kits introduced, treatment regimen, and vector control strategies in the study area.

This study found progressive improvement in performance of older VMWs. With a qualifying score of 70%, 100% qualifying rates were achieved and significant improvement in scores using the 30-point monitoring checklist and ‘shadowing’ of workers before formal training. The best performers amongst the 260-field staff were recognized at the national-level Malaria Elimination Advisory Group (MEAG) meetings as ‘VMW/MFC of the year’ [15]. These awards were instituted to instill confidence in the field workers for their contributions.

The improvement in scores was seen in 95% of the candidates. Absence of improvement in 4% candidates may be attributed to having prior experience in malaria projects and scoring high in both pre- and post-trainings, hence, no difference in scores was observed. The mystery of 1% candidates who deteriorated after trainings could be attributed to fluke responses given by them in the pre-trainings, which actually helped them score higher than trained responses in post-training assessment.

The caste distribution of candidates compliments the socio-demographics data collected by MEDP for Mandla district. However, the Scheduled Tribes representation of candidates is lesser than the district-wide average. This might be due to the lesser number of candidates from this particular group who applied for the posts in the project. It should be noted that the eligibility for the job of a Village Malaria Worker was grade 12, yet more than half of the applicants were college graduates.

Madhya Pradesh has the highest proportion of tribal population in the country [20]. The inability to recruit due to absence of trained workforce in tribal regions of India and South-East Asia has been documented before [20, 21]. Prior studies have revealed that the barriers for creating trained and educated workforce include vacant teaching positions, lack of infrastructure, lack of foresightedness, medium (language) of study, inadequate family support, and economic conditions. However, In Mandla, the challenge was not the lack of qualified workforce, but quality of education they have received. Post-graduates apply for jobs which require high-school diplomas as the minimum qualification, but are often unable to qualify the basic screening tests. Overqualified candidates applying for such jobs could be because of lack of quality education. Similar concerns have been raised by studies in Kochi and West Bengal [22, 23].

Most of the job vacancies in Mandla district belong in the public-sector. These jobs demand a certain certificate or degree which can be obtained through open-schooling systems. These systems offer negligible classroom-presence and open-book examinations. While there are gains on quantitative literacy indicators, there is very little focus on qualitative literacy. This issue has also been raised in the India Education Report 2002 [24].

This study observed a slight improvement in the scores between 2017 and 2018 (2017 cohort), but significant improvement was seen in the same cohort in the 2019 review. The reason behind this improvement may be attributed to the introduction of the 30-point monitoring checklist in the month of March 2019. This comprehensive checklist was regularly administered by the Malaria Field Coordinators (MFC) during each visit to their Village Malaria Workers (VMW). On an average, each MFC administered two checklists for two VMWs every day. Prior to this tool, the supervision was done without a pre-set checklist. The tool facilitated a more structured process with robust monitoring, evaluation, and learning on a periodic basis.

While the attributed reason behind success of the 2017 cohort in the 2019 review is justified, there was also significant improvement in scores of fresh candidates of 2019 as compared to the scores of fresh candidates of 2018 and 2017. They were not monitored using the checklist. Instead, a new strategy known as ‘Shadowing’ was introduced in February 2019.

As part of Shadowing, the newly selected candidates were given a choice of field posting. Following which, they were deployed in their respective fields and were asked to accompany their MFC. For 15 days, the new VMWs observed and learned by accompanying their supervisor. Since they were untrained and uncertified, they did not perform any diagnosis and treatment. Following this exercise, the VMWs who were ready to accept the postings were formally appointed and trained under the project. There was a significant improvement in attitude, knowledge and perceptions of the VMWs who were part of the ‘shadowing’ programme, and hence, the same was reflected in their training scores.

The difference between mean scores of 12th grade and college graduates were significant during their first trainings. However, the difference was much less (0.56) after their second trainings. The reduction in difference could be because of the acquired work-experience. Looking longitudinally, the technical capabilities of both groups irrespective of their educational qualification were at-par with each other. Introduction of monitoring checklist and ‘shadowing’ further contributed to reduction in this difference. It was observed that the oldest cohort (2017) of Village Malaria Workers continued to show improvement in performance and were the highest scorers in the last training conducted in 2019. The oldest Village Malaria Workers have proven to be the best performers in other studies as well [25].

The test was divided into three major themes—general knowledge related to malaria, diagnosis and treatment and vector control. Progressive improvement in all thematic areas was seen from 2017 to 2019. Similar improvement in all these thematic areas was seen in a post-training assessment conducted on Village Malaria Workers of Cambodia [26]. The weakest thematic area which continued to be a bottle-neck was vector control and word-based problems in diagnosis and treatment. The names of mosquitos and technical guidelines for Indoor Residual Sprays were answered incorrectly maximum number of times. Other lacunae were the case study-like questions about malaria cases asked in the question paper. These questions were attempted by everyone but only a few answered the complete management of the case. These areas will be better taught using hand-holding during their regular fieldwork and practical demonstrations. The Cambodia study also found vector ecology as the most difficult subject-area for the Village Malaria Workers [26].

In conclusion, the MEDP has successfully demonstrated the need for high standards in recruitment, training, performance, and assessment of field staff involved in surveillance and case management, IEC/BCC, vector control, and capacity building. The project achieved a reduction of 91% indigenous malaria cases within 31 months of its operations, which was in-part due to the skilled field workforce [27]. This makes the Village Malaria Workers under the supervision of Malaria Field Coordinators as the backbone of the project. Since, these would be temporary workers in several national and sub-national malaria elimination programmes, the trained workers of MEDP and similar projects are not only of high-value for the malaria elimination initiatives, but also for other public health delivery systems of national importance. The training modules developed by MEDP, as well as learnings from it could be used in Indian national malaria elimination programme as well as similar programmes around the world.

## Supplementary Information

**Additional file 1: Annexure S1.**

**Additional file 2: Annexure S2.**

## Data Availability

We have reported all the findings in this manuscript. The hardcopy data is stored at MEDP Office in Mandla, Madhya Pradesh and Indian Council of Medical Research-National Institute of Research in Tribal Health (ICMR-NIRTH), Jabalpur, Madhya Pradesh. Softcopy data is available on the project server of MEDP hosted by Microsoft Azure. If anyone wants to review or use the data, they should contact: Dr. Altaf A. Lal—Project Director – Malaria Elimination Demonstration Project, Mandla; Foundation for Disease Elimination and Control of India, Mumbai, India 482003. E mail: altaf.lal@sunpharma.com.

## Abbreviations

DXRX: Diagnosis and treatment
KAP: Knowledge, attitude, and perceptions
MEDP: Malaria Elimination Demonstration Project
MFC: Malaria Field Coordinator
OBC: Other Backward Caste
PVC: Vector control
SC: Scheduled Caste
ST: Scheduled Tribe
VMW: Village Malaria Worker
