# Supplementary material for: Significance of training, monitoring and assessment of malaria workers in achieving malaria elimination goal of Malaria Elimination Demonstration Project
Source: Malar J. 2021 Jan 7;20:27. doi: 10.1186/s12936-020-03534-9 (PMC7789890; doi:10.1186/s12936-020-03534-9)
Supplement: Supplementary file 1 — Additional file 1: Annexure S1. [file 12936_2020_3534_MOESM1_ESM.docx]

**Malaria Elimination Demonstration Project, Mandla, Madhya Pradesh**

Foundation for Disease Elimination and Control of India (FDEC – India)

Pre and Post-test questionnaire

1. How many community health centres (CHC) are there in the Mandla district?
2. 6
3. 8
4. 9
5. 10
6. What is Malaria?
7. Fever
8. Communicable diseases
9. Bacteria
10. Virus
11. How does malaria spread?
12. Contaminated air
13. Contaminated water
14. Mosquito
15. From contaminated food
16. Where do mosquitoes that spread malaria thrive?
17. In Unclean water
18. In stagnant clean water
19. In flowing water
20. In the mud
21. Outside of the Africa continent which country has recorded the highest number of deaths due to malaria?
22. Sri Lanka
23. India
24. China
25. America
26. When does ACT + PQ have to be taken?
27. On having fever
28. Simple malaria
29. When pf malaria is confirmed
30. On having viral fever
31. What is the goal of the MEDP Mandla Project?
32. Malarial Elimination
33. Malaria Eradication
34. Malaria Control
35. How many types are there of parasites of malaria?
36. 4
37. 6
38. 5
39. 2
40. When an infected female mosquito bites a healthy human, then …………… enters the human body with saliva.
41. Bacteria
42. Germ
43. Parasite
44. What to do in case of high fever with severe cold, headache, and vomiting?
45. Should be taken immediately to the nearest any unregistered doctor
46. Should be taken immediately to the nearest priest/panda for exorcism
47. Should be taken immediately to the nearest government health worker and do a blood test by the Rapid Diagnostic Test (RDT) kit.
48. When do mosquitoes that spread malaria suck human blood?
49. In the morning
50. In the afternoon
51. In the evening
52. After sleeping at night
53. Which is the easiest way to prevent from malaria-spreading mosquitoes?
54. Applying mosquito net
55. Smoke in the evening
56. Spraying insecticides at home
57. All of the above
58. Why do not spray the wall and kettle shed outside the house?
59. By doing this, outside mosquitoes will come inside and harm
60. Both animals and humans will be harmed
61. Outer walls will be damaged
62. Which group does Malaria affect the most?
63. 05 to 10 year old child
64. Elderly people above 60 years
65. New-born and pregnant women
66. All of the above
67. What tests are done to confirm the malaria parasite in the blood?
68. Blood slide test by microscope
69. By bivalent rapid diagnostic test kit
70. QBC test
71. ELISA test
72. All of the above
73. After how long can one see the result of the bivalent rapid diagnostic test?
74. 1-2 minute
75. 5-10 minute
76. 15-30 minute
77. 30-60 minute
78. Write the name of the disease spread by mosquito.
79. ……………………………………………………………….
80. ……………………………………………………………….
81. ……………………………………………………………….
82. Write the names of three mosquitoes that spread malaria.
83. …………………………………………………………………
84. …………………………………………………………………
85. …………………………………………………………………
86. Write the names of three parasites of malaria.
87. …………………………………………………………………
88. …………………………………………………………………
89. …………………………………………………………………
90. Which five colours does the ACT blister packet come age-wise?
91. Red, Blue, Black, Yellow, Purple
92. Pink, Blue, Black, Red, White
93. Pink, Yellow, Green, Red, White
94. Red, Blue, Black, Pink, Purple

Name: - …………………………………………………………

Village: - ………………………………………………………

Block: - …………………………………………………………

Mobile No.:-

Mobile No.:-

Signature
